# From Molecular Silence to Lymphoid Blast Phase: Diagnostic and Therapeutic Challenges in a Young Female Patient With Chronic Myeloid Leukemia

**DOI:** 10.7759/cureus.103856

**Published:** 2026-02-18

**Authors:** Zainab H Alqallaf, Sara Atwa

**Affiliations:** 1 Medical Oncology, Faculty of Medicine, Mansoura University, Mansoura, EGY

**Keywords:** asciminib, b-lymphoblastic phase, chronic myeloid leukemia (cml), molecular silence, tki intolerance, tyrosine kinase inhibitor (tki)

## Abstract

Chronic myeloid leukemia (CML) is a triphasic myeloproliferative neoplasm characterized by the breakpoint cluster region-Abelson BCR::ABL1 fusion gene, typically detected by reverse transcription-quantitative polymerase chain reaction (RT-qPCR). We present a case of a 20-year-old female patient who presented with non-specific constitutional symptoms and was diagnosed with CML based upon detection of the Philadelphia (Ph) chromosome by fluorescence in situ hybridization (FISH), while repeated molecular testing remained negative. Notably, during treatment with tyrosine kinase inhibitors (TKIs), she became intolerant to first- and second-generation TKIs, including the branded and generic imatinib, nilotinib, and dasatinib, followed by progression into lymphoid blast phase. This case highlights the diagnostic challenges and therapeutic complexity of managing CML in the setting of multi-TKI intolerance. Importantly, it underscores the persistent molecular silence despite repeated RT-qPCR testing and the successful introduction of asciminib as a novel therapeutic alternative.

## Introduction

Chronic myeloid leukemia (CML) is a common triphasic myeloproliferative neoplasm characterized by uncontrolled proliferation of leukemic cells of myeloid origin [1]. This proliferation typically manifests as a full spectrum of granulocytic precursors, including blasts, promyelocytes, myelocytes, and metamyelocytes, along with a marked left shift, significant basophilia, and mild eosinophilia [2]. Although it primarily affects older populations, adolescents and young adults (AYAs) represent a small subgroup who tend to experience a more aggressive disease course [3]. The uncontrolled proliferation of leukemic cells is driven by the activity of the tyrosine kinase of the breakpoint cluster region-Abelson BCR::ABL1 fusion gene, which arises from t(9;22)(q34;q11.2) translocation, also known as the Philadelphia (Ph) chromosome. The translocation can be detected by fluorescence in situ hybridization (FISH), and the corresponding transcript can be identified by reverse transcription-quantitative polymerase chain reaction (RT-qPCR), which is applicable for the diagnosis of a typical CML case. Tyrosine kinase inhibitors (TKIs) remain the cornerstone of frontline treatment for CML [1]. This report presents a case of CML in a young adult with lymphoid blast phase transformation, highlighting diagnostic challenges related to molecular-cytogenetic discordance and the potential role of asciminib in advanced-phase disease, particularly in the context of multi-TKI intolerance.

## Case presentation

A previously healthy 20-year-old female patient from North Africa was referred to the outpatient clinic. Her past medical and family history was unremarkable. The patient reported unintentional weight loss and generalized fatigue for a few weeks. On physical examination, she was clinically stable, with mild to moderate splenomegaly, no hepatomegaly, and no lymphadenopathy. Initial laboratory findings are summarized in Table 1.

**Table 1:** Serum laboratory investigation at the time of diagnosis. WBC: white blood cell, Hb: hemoglobin, SGPT: serum glutamic-pyruvic transaminase, ALT: alanine aminotransferase, SGOT: serum glutamic-oxaloacetic transaminase, AST: aspartate aminotransferase, PT: prothrombin time, ESR: erythrocyte sedimentation rate, LDH: lactate dehydrogenase

Investigation	Value	Reference Range	Interpretation
WBC count	260 ×10⁹/L	4-11 ×10⁹/L	High (leukocytosis)
Platelet count	927 ×10⁹/L	150-450 ×10⁹/L	High (thrombocytosis)
Hb	7.2 g/dL	12.0-16.0 g/dL	Low (anemia)
SGPT/ALT	14 U/L	30-65 U/L	Normal
SGOT/AST	18 U/L	15-37 U/L	Normal
Serum total bilirubin	0.5 mg/dL	0.3-1.0 mg/dL	Normal
PT	14.8 seconds	11.0-15.0 seconds	Normal
ESR, first hour	80 mm/hour	<10 mm/hour	High
LDH	1334 U/L	100-190 U/L	High
Creatinine	0.8 mg/dL	0.6-1.3 mg/dL	Normal

Peripheral blood film demonstrated a significant granulocytic left shift with eosinophilia, basophilia, and 2% circulating blasts. Abdominal ultrasound revealed splenomegaly measuring 19 cm.

She was admitted and started on hydroxyurea for cytoreduction along with supportive care. Follow-up complete blood count (CBC) showed improvement, with a decrease in white blood cell (WBC) count to 17 ×10^9^/L, platelet count to 462 ×10^9^/L, and an increase in hemoglobin (Hb) to 11.1 g/dL.

RT-qPCR for BCR::ABL1 p210 transcript was performed on peripheral blood one day before cytoreduction with hydroxyurea and unexpectedly showed no detectable transcript (detection limit: 0.000%). The detection limit was defined according to our laboratory’s established cut-off, which ranges from 0.001% to 0.0001%, and validation was performed using positive and negative controls in each run.

However, FISH analysis was performed on interphase nuclei from peripheral blood using a BCR/ABL dual-color, dual-fusion probe set (Cytocell, Cambridge, UK). A total of 200 interphase nuclei were evaluated, demonstrating positivity for t(9;22)(q34;q11.2) in 55% of nuclei (110/200), confirming the presence of the Ph chromosome. Conventional metaphase cytogenetic analysis of the bone marrow was not performed; therefore, the presence of additional cytogenetic abnormalities (ACAs) could not be assessed. Testing for the Janus kinase 2 (JAK2) mutation was negative.

Due to the discordance between molecular testing and cytogenetic results, both RT-qPCR and FISH were repeated. FISH once again confirmed the presence of t(9;22), whereas RT-qPCR for BCR::ABL1 p210 remained negative. Nilotinib was initiated at a dose of 300 mg twice daily (BID). However, it was discontinued due to a drug-induced hypersensitivity reaction.

Additional testing for RT-qPCR for BCR::ABL1 p190 transcript was performed on peripheral blood approximately two months after cytoreduction and showed no detectable transcript. Testing for the p230 transcript was likewise negative.

The RT-qPCR analysis was performed using the Ipsogen BCR-ABL1 Mbcr IS-MMR Kit (QIAGEN, Venlo, The Netherlands) on a DNA technology real-time PCR thermal cycler (QIAGEN, Venlo, The Netherlands) to detect BCR-ABL1 p210 (b2a2, b3a2) and p190 (e1a2) transcripts. BCR::ABL1 p230 testing was conducted at an external laboratory. Evaluation for atypical BCR::ABL1 transcripts (beyond the common p210/p190/p230), including multiplex PCR or RNA-fusion/next-generation sequencing (NGS) testing, was not performed and represents a limitation of this report.

**Figure 1:**
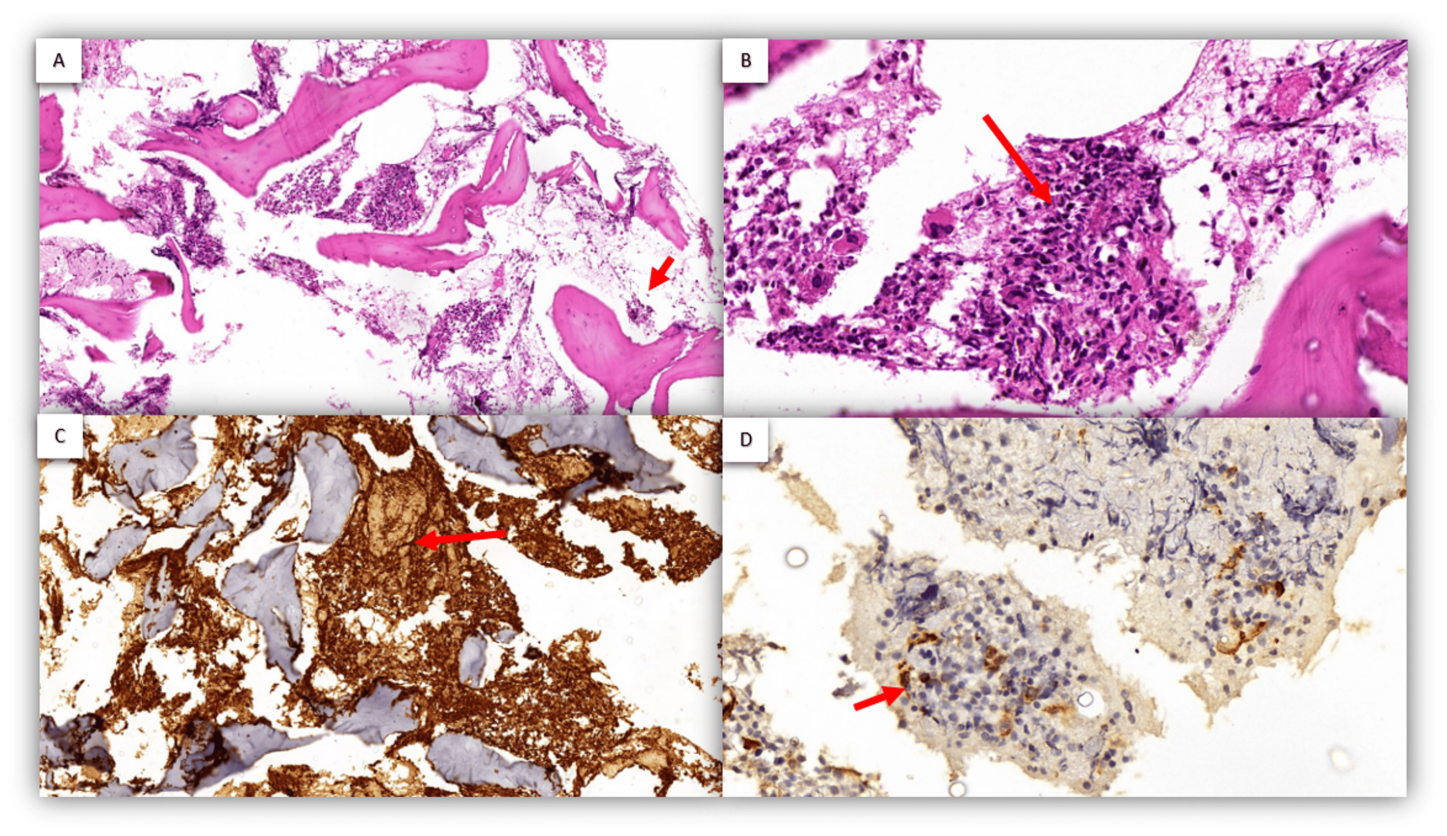
Initial bone marrow biopsy. (A) H&E stain shows a washed-out bone marrow space (arrow). (B) H&E stain demonstrates an increased myeloid series (arrow). (C) MPO stain highlights increased myeloid series (arrow). (D) Immunohistochemistry for CD34 shows blast cells <5% (arrow). H&E: hematoxylin and eosin, MPO: myeloperoxidase

Bone marrow biopsy revealed a hypercellular marrow with marked myeloid hyperplasia, highlighted by myeloperoxidase (MPO) positivity in 80-90% of cells. CD34 and CD117 positivity in scattered cells (<5%) marked erythroid suppression and decreased megakaryocytes. Findings were consistent with chronic-phase CML. Bone marrow biopsy findings are shown in Figure 1.

The patient was started on branded imatinib 400 mg daily, which was initially well tolerated. However, five months later, after switching to a generic formulation of imatinib, the patient developed recurrent episodes of vomiting, resulting in poor adherence and subsequent discontinuation of therapy for two months.

She was re-admitted with a CBC showing WBC count of 345 ×10^9^/L, platelet count of 665 ×10^9^/L, and Hb of 8.9 g/dL. Dasatinib was introduced; however, the patient developed shortness of breath. Chest computed tomography (CT) revealed mild bilateral pleural effusions with interstitial pneumonitis superimposed on an atypical pulmonary infection. She was stabilized and eventually discharged on branded imatinib.

Given the patient’s intolerance to both first- and second-generation TKIs, human leukocyte antigen (HLA) matching was initiated in preparation for allogeneic hematopoietic stem cell transplantation (HSCT). Branded imatinib was continued as the only tolerated TKI while awaiting a matched donor.

During follow-up while on imatinib, the CBC revealed marked leukocytosis (WBC count of 102 ×10^9^/L), thrombocytosis (platelet count of 568 ×10^9^/L), and anemia with Hb of 9.8 g/dL. She was started on subcutaneous cytarabine (Ara-C) as a potent cytoreductive therapy, with the goal of avoiding tumor lysis syndrome by reducing WBC count below 100 ×10^9^/L while awaiting definitive therapy.

A repeat bone marrow examination showed 20% blasts. Flow cytometry revealed 21% lymphoblasts expressing CD19, CD10, CD34, CD33, and dim CD45, and negative for CD7, CD13, CD117, and HLA-DR, consistent with B-lymphoblastic transformation. Cytogenetic analysis once again confirmed the presence of the Ph chromosome, while RT-qPCR for BCR::ABL1 p190 transcript remained negative, supporting a Ph+ B-lymphoid blast phase arising from CML.

The diagnosis of CML in lymphoid blast phase rather than de novo Ph+ acute lymphoblastic leukemia (ALL) was supported by well-documented chronic-phase features at initial presentation, including splenomegaly, granulocytic left shift, basophilia, eosinophilia, and bone marrow morphology consistent with chronic-phase CML. Cytogenetic analysis demonstrated BCR::ABL positivity by FISH in approximately 50% of analyzed nuclei at diagnosis. At transformation, bone marrow examination showed features consistent with lymphoid blast phase, and repeat FISH testing remained positive. Conventional karyotyping was not performed, and the exact percentage of FISH positivity at transformation could not be retrieved due to sample unavailability.

The patient was started on an augmented Berlin-Frankfurt-Münster (BFM) chemotherapy protocol, typically used for young patients with ALL, beginning with the induction phase, during which she achieved complete remission. Post-induction bone marrow was mildly hypercellular for age, with blasts less than 5%, consistent with chronic-phase CML. Following B-lymphoblastic transformation, allogeneic HSCT remained the intended definitive therapy; however, despite an early and comprehensive donor search, no suitable HLA-matched donor was identified.

Asciminib was introduced at a dose of 40 mg BID, chosen for its better tolerability, as the patient had a history of TKI intolerance except for branded imatinib. This dose is approved for the chronic-phase CML; however, no data are available regarding the appropriate dose in the blast phase, so it was selected for this patient. Asciminib was initiated once it became available, starting from the consolidation phase, and the WBC count normalized following administration. The patient also received prophylactic cranial and spinal radiotherapy with a total dose of 24 Gray (Gy) over 12 fractions. To improve clarity and interpretability, the patient’s treatment course is summarized in a concise timeline (Table 2).

**Table 2:** Timeline of therapeutic interventions according to disease phase. WBC: white blood cell, CML: chronic myeloid leukemia, TKI: tyrosine kinase inhibitor, Ara-C: cytarabine, BFM: Berlin-Frankfurt-Münster, ALL: acute lymphoblastic leukemia

Phase/Clinical Stage	Therapy	Reason for Use/Change	Response/Toxicity
Initial presentation	Hydroxyurea	Cytoreduction	WBC count decreased to 17 ×10⁹/L
Chronic-phase CML	Nilotinib	First-line second-generation TKI	Hypersensitivity reaction leading to discontinuation
Chronic-phase CML	Branded imatinib	Switched due to nilotinib intolerance	Well tolerated
Chronic-phase CML	Generic imatinib	Switch to a generic formulation	Repeated vomiting leading to treatment discontinuation
Chronic-phase CML	Dasatinib	Switched due to intolerance to prior TKIs	Shortness of breath, mild pleural effusion with interstitial pneumonitis
Chronic-phase CML	Branded imatinib	Only tolerated TKI	Well tolerated
Suspicion of blast phase	Subcutaneous Ara-C	Cytoreduction and prevention of tumor lysis syndrome	Persistent leukocytosis with subsequent confirmation of lymphoid blast transformation
B-lymphoblastic phase	Augmented (BFM) chemotherapy protocol	Chemotherapy protocol used for young patients with ALL	Complete remission
Consolidation	Asciminib	Initiated due to prior TKI intolerance	WBC count normalized

## Discussion

Several BCR::ABL1 fusion transcripts can arise from different BCR gene breakpoints, producing fusion proteins of varying molecular weights. The major breakpoint (M-bcr) produces two common variants, e13a2 and e14a2, both encoding the p210 fusion protein. The minor breakpoint (m-bcr) results in the e1a2 transcript, which encodes the p190 protein. The micro breakpoint (u-bcr) produces the e19a2 transcript, resulting in the p230 protein. Rare atypical transcripts can also occur, including e1a3, e13a3, e14a3, e19a3, e6a2, and e8a2, which occur outside the common BCR and ABL1 intron-exon boundaries [4]. 

It is important to note that more than 95% of CML cases are Ph chromosome-positive [2]. In CML cases where the Ph chromosome is not detected by conventional metaphase karyotyping from bone marrow, FISH on interphase nuclei or RT-qPCR can confirm its presence. Rare BCR::ABL1 variants may require multiplex assays or NGS [2].

AYAs, typically defined as patients aged 15-29 years, represent a small subset of CML cases, with a median age at diagnosis around 65 years for the overall population [3,5]. While AYAs generally do not show significantly worse progression-free survival (PFS) or overall survival (OS), they often present with aggressive disease features, including high WBC and blast counts, lower Hb, and more pronounced splenomegaly, which may impact short-term outcomes [3]. Generally, outcomes of patients with CML have improved since the approval of TKIs. The rate of progression to advanced phase-CML is now low and often related to TKI intolerance or failure [6]. TKI intolerance may be related to both on-target and off-target effects, affecting various organ systems with varying severity, and is a major reason for therapy discontinuation [7].

Generic imatinib is a cost-effective alternative to branded imatinib for treating CML. Although some studies suggest variability, most evidence supports similar efficacy and toxicity compared to the branded version. However, continued research is needed to assess long-term outcomes [8].

A significant complication of CML is transformation into the lymphoid blast phase. Defined by a 20% or more increase in blast cells either in bone marrow or peripheral blood, it causes significant impairment in the disease outcome [9]. Lymphoid blast phase accounts for about 30% and is less common than the myeloid blast phase [9]. B-lymphoid blast phase is more common than T-lymphoid blast phase and carries a poor prognosis. Treatment of lymphoid blast phase in CML mainly involves high-intensity chemotherapy protocols like those used for Ph+ ALL, especially in young patients with excellent performance status. This is typically combined with second or third-generation TKIs or HSCT rather than imatinib or TKI monotherapy [9,10].

Asciminib, a novel specifically targeting the ABL myristoyl pocket (STAMP) inhibitor of BCR-ABL, binds to the allosteric site rather than the adenosine triphosphate (ATP)-binding site targeted by traditional TKIs [5]. In the ASCEMBL trial, asciminib was shown to be a durable and well-tolerated treatment, with few patients discontinuing therapy because of side effects. Most hematological and non-hematological adverse events (AEs) occurred early in the treatment course, were generally not severe, and were manageable. Asciminib demonstrated superior efficacy with an increasing rate of deep molecular response (DMR) over time [11]. Preclinical studies also support its activity in CML blast phase and Ph+ ALL, showing leukemic stem cell suppression and hematopoietic recovery in murine models, suggesting its potential as monotherapy or in combination [10]. A recent case reported successful use of asciminib 200 mg BID in the consolidation phase after ponatinib failure and induction chemotherapy, resulting in complete molecular response in a patient with myeloid blast crisis harbouring the T315I mutation [12].

In our case, the use of asciminib in lymphoid blast phase CML in the setting of TKI intolerance suggests a potential expansion of its clinical applicability beyond its established use in TKI-resistant disease. 

## Conclusions

Clinicians should maintain a high index of suspicion when encountering complex cases of CML, particularly in young adults, and should recognize the importance of integrating clinical, molecular, and histopathological data in the diagnosis of atypical variants. Emphasis should also be placed on comprehensive molecular testing strategies and individualized therapeutic approaches in young patients with CML. Asciminib may represent a well-tolerated therapeutic option in advanced-phase CML with intolerance to multiple TKIs.

## References

[1] Jabbour E, Kantarjian H (2024). Chronic myeloid leukemia: 2025 update on diagnosis, therapy, and monitoring. Am J Hematol.

[2] Osman AE, Deininger MW (2021). Chronic myeloid leukemia: modern therapies, current challenges and future directions. Blood Rev.

[3] Iezza M, Cortesi S, Ottaviani E (2023). Prognosis in chronic myeloid leukemia: baseline factors, dynamic risk assessment and novel insights. Cells.

[4] Liu B, Zhang W, Ma H (2016). Complete cytogenetic response to nilotinib in a chronic myeloid leukemia case with a rare e13a3(b2a3) BCR-ABL fusion transcript: a case report. Mol Med Rep.

[5] Kalmanti L, Saussele S, Lauseker M (2014). Younger patients with chronic myeloid leukemia do well in spite of poor prognostic indicators: results from the randomized CML study IV. Ann Hematol.

[6] Choi EJ (2023). Asciminib: the first-in-class allosteric inhibitor of BCR::ABL1 kinase. Blood Res.

[7] Shyam Sunder S, Sharma UC, Pokharel S (2023). Adverse effects of tyrosine kinase inhibitors in cancer therapy: pathophysiology, mechanisms and clinical management. Signal Transduct Target Ther.

[8] Erçalışkan A, Seyhan Erdoğan D, Eşkazan AE (2021). Current evidence on the efficacy and safety of generic imatinib in CML and the impact of generics on health care costs. Blood Adv.

[9] Yohannan B, George B (2022). B-lymphoid blast phase-chronic myeloid leukemia: current therapeutics. Int J Mol Sci.

[10] Chatain N, Baumeister J, Szymanski de Toledo MA (2024). Asciminib antagonizes transplantable BCR::ABL1-positive lymphoid blast crisis in vivo by targeting malignant stem cells. Leukemia.

[11] Hochhaus A, Réa D, Boquimpani C (2023). Asciminib vs bosutinib in chronic-phase chronic myeloid leukemia previously treated with at least two tyrosine kinase inhibitors: longer-term follow-up of ASCEMBL. Leukemia.

[12] Tomassetti S, Lee J, Qing X (2022). A case of chronic myelogenous leukemia with the T315I mutation who progressed to myeloid blast phase and was successfully treated with asciminib. Clin Case Rep.

